# Integrative analysis identifies CXCL11 as an immune-related prognostic biomarker correlated with cell proliferation and immune infiltration in multiple myeloma microenvironment

**DOI:** 10.1186/s12935-022-02608-9

**Published:** 2022-05-14

**Authors:** Huizhong Wang, Ruonan Shao, Wenjian Liu, Shumei Peng, Shenrui Bai, Bibo Fu, Congling Zhao, Yue Lu

**Affiliations:** 1 Sun Yat-sen University Cancer Center, 651 Dongfeng East Road, Guangzhou, Guangdong, 510060 China; 2 State Key Laboratory of Oncology in South China, 651 Dongfeng East Road, Guangzhou, Guangdong, 510060 China; 3 Collaborative Innovation Center for Cancer Medicine, 651 Dongfeng East Road, Guangzhou, Guangdong, 510060 China; 4 Department of Pediatrics, Guangdong Women and Children Hospital, Guangzhou, 510060 China

**Keywords:** Multiple myeloma, Prognostic model, CXCL11, Macrophages, Tumor microenvironment

## Abstract

**Purpose:**

The interaction between tumor cells and tumor microenvironment (TME) has an important impact on progression and prognosis of multiple myeloma (MM), and has been proven to be promising therapeutic targets. This study intended to explore the relationship between TME and prognosis and identify valuable biomarkers of MM.

**Methods:**

The transcriptomic and clinical information of MM retrieved from the Gene Expression Omnibus (GEO) were used to establish the model. The curve of Kaplan–Meier survival and the time-dependent receiver operating characteristic (ROC) were used to appraise the predictive ability. A nomogram was established for clinical application. Furthermore, the CIBERSORT algorithm was used to investigate the relation between IRGPI with the infiltration of immune cells. We also used histology, as well as in vitro and in vivo experiments to validate these findings.

**Results:**

The results demonstrated an immune-related gene-based prognostic index (IRGPI) combined with clinical information. Patients were separated into high- and low-risk groups based on risk score, which had significantly difference in survival status and immune infiltrations. Furthermore, we identified CXCL11 as a key factor, which positively promotes the progression of MM and correlate with macrophage M2-like polarization and tumor immune cells infiltration.

**Conclusion:**

Our findings suggest the IRGPI significantly demonstrate the differential prognosis and prediction of immune cells infiltration. It provides some insights into the complex interaction between myeloma tumor cells and the TME, as well as in the development of a novel biomarker target for anti-MM therapy.

**Supplementary Information:**

The online version contains supplementary material available at 10.1186/s12935-022-02608-9.

## Introduction

Multiple myeloma (MM) is a clonal proliferative heterogeneous disorder of plasma cells. Among all hematological malignancies, it accounts for an almost 17% prevalence rate, with an increasing incidence, globally [1, 2]. As a kind of malignancy, where a preferential localization of clonal plasma cells occurs in bone marrow (BM). The MM cells proliferation and the altered BM microenvironment suppress immunity and evade immune surveillance[3]. The impact of tumor genetic composition on the tumor microenvironment (TME) will help to provide diagnostic and prognostic biomarkers for MM patients, as well as novel therapeutic targets for therapy [4–7].

Immunotherapy uses a variety of immune cells either to suppress or kill tumor cells, lowering the incidence of tumor recurrence and metastasis [8, 9], on the other hand, can only benefit a small number of patients. Recent investigations have indicated that TME, mainly composed of stromal cells, immune/inflammatory cells, and their corresponding cytokines, plays a significant role in tumor progression, development, immune evasion, and treatment resistance [10, 11]. Studies have confirmed that the types and proportions of immune cells in TME may influence both treatment response and clinical outcome [12]. Immune-related genes (IRGs) have been increasingly confirmed to have a vital role in different kinds of malignancies [13–15]. The biomarkers related to TME are potential in the prediction, treatment, and prognosis of cancers [16, 17]. Therefore, the analysis of the complex interaction between them will help to provide novel avenues for clinical application.

Tumor-associated macrophages (TAMs) are the key components of TME. A high extent of TAMs infiltration is related to poor disease prognosis in many types of tumors, drawing attention towards their prognostic relevance [18–21]. TAMs are usually differentiated into either the anti-tumor M1 subtype or tumor-promoting M2 subtype. It is generally considered that M2 has poor reactive nitrogen and antigen presentation ability, which can inhibit anti-tumor immunity and promote tumor progression [22, 23]. Furthermore, TAMs in the body are largely polarized to M2-like phenotypes in advanced stages of cancer [24]. Evidences showed that increased levels of M2 macrophages in bone marrow microenvironment were also revealed to tuomor progression and poor prognosis in MM [25, 26]. Therefore, we have focused on the the relationship between macrophage and MM cells and selected M2 macrophage as our cell model in our verification study.

In this study, we firstly investigated the prognostic significance of IRGs family members in MM to develop an individualized model, and further identified CXCL11 as a key regulator. As a potential therapeutic target which linked to the recruitment and infiltration of macrophages in TME, the expression of CXCL11 and its biological roles were further investigated and explored in vitro and in vivo functional experiments. In general, these findings suggested the potential prognostic significance of CXCL11 in TME, which may help to provide a potential target for the prognosis prediction and therapy of MM in the future.

## Materials and methods

### Patients and clinical samples

**Fig. 1:**
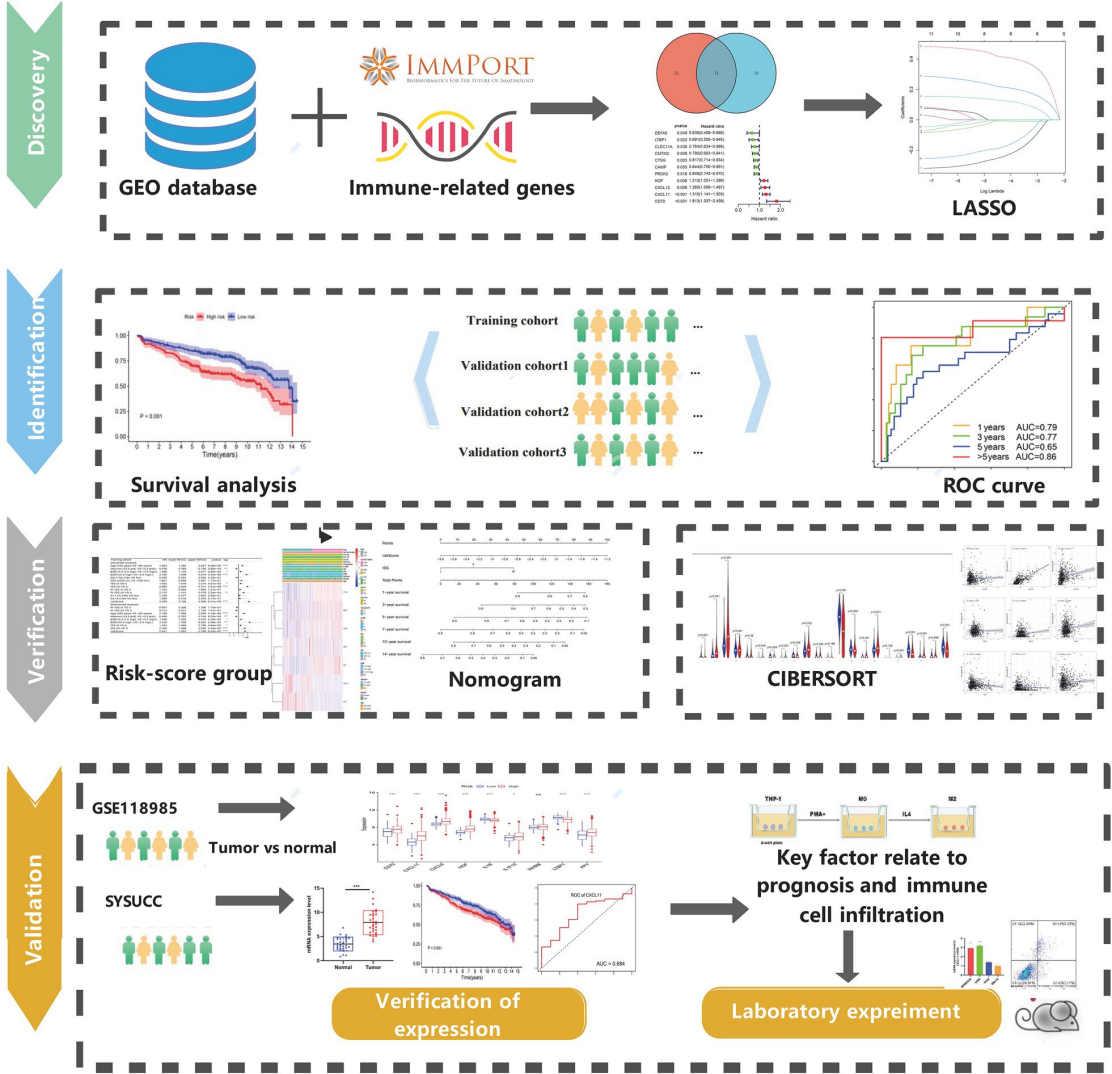
Flow chart of the evaluation and selection of IRGPI

The RNA profiles and related clinical information of all cohorts were gathered from the Gene Expression Omnibus (GEO) databases: GSE136324, GSE57317, and GSE4581 cohorts. Samples were excluded if available survival data were lacking. Additional file 5: Table S1 provides a comprehensive description of patient characteristics in the cohorts. The IRGs were acquired from the ImmPort dataset (https://www.immport.org/home). The relative gene expression was normalized using the “limma” R package. A workflow chart describing the samples utilized at each stage of analysis is presented in Fig. 1. Furthermore, GSE118985 and our own cohort were selected to help verify and screen the key factor. The 30 MM samples (newly diagnosed with MM and normal samples)were obtained from the Sun Yat-sen University Cancer Center (SYSUCC). The study was conducted after approval through SYSUCC 's internal review and ethics boards.

### Identification and verification of the prognostic signature

The GSE136324 cohort was segregated into training cohort and validation cohort according to the ratio of 4:1.GSE57317 and GSE4581 cohorts were chosen as external validation cohorts. Initially, univariate Cox regression was used to look for IRGs with prognostic value (p < 0.05) due to the survival data, and a venn diagram was generated to visualize the intersections of the three cohorts. Cancer Cell Line Encyclopaedia (CCLE) was used to further confirm these prognostic-related IRGs at the cellular level.The least absolute shrinkage and selection operator (LASSO) Cox regression assessment was used for ascertaining the most suitable weighting coefficient for the IRGs [27]. Next, the maximum likelihood estimator was penalized using tenfold cross-validation. Furthermore, the essential penalty parameter λ values were identified using the penalized maximum likelihood estimator's minimum criteria. Finally, a general formula based on the training cohort was employed for the calculation of the metabolic risk score. Accordingly, the patients were assigned into two groups, namely high risk (HR) and low risk (LR) (Additional file 1: Fig. S1).

**Fig. 2:**
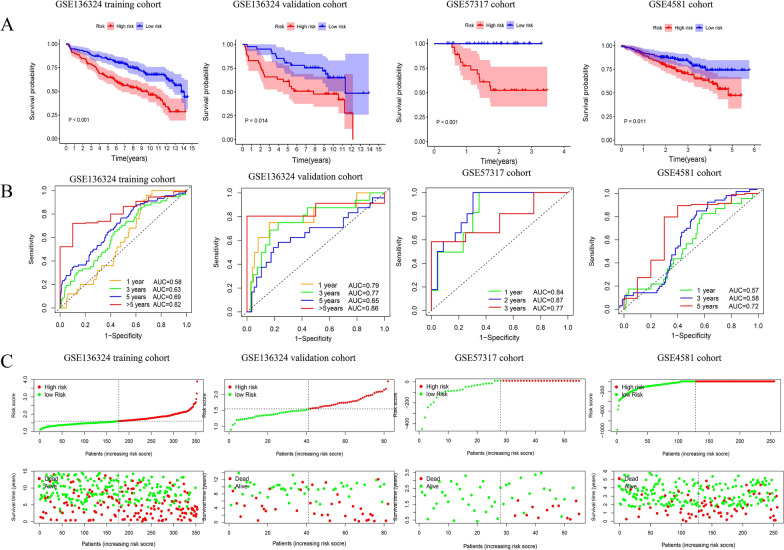
Time-dependent ROC analysis, survival outcome analysis and Kaplan- Meier analysis and risk score analysis for the IRGPI accurately predicts survival of MM patients in LR and HR. **A** Kaplan–Meier curve of the prognostic model in the training cohort the validation cohorts, **B** Time-dependent ROC curves analyses of the model in all cohorts, **C** Risk score distribution of the prognostic model on the training cohort the validation cohorts

**Fig. 3:**
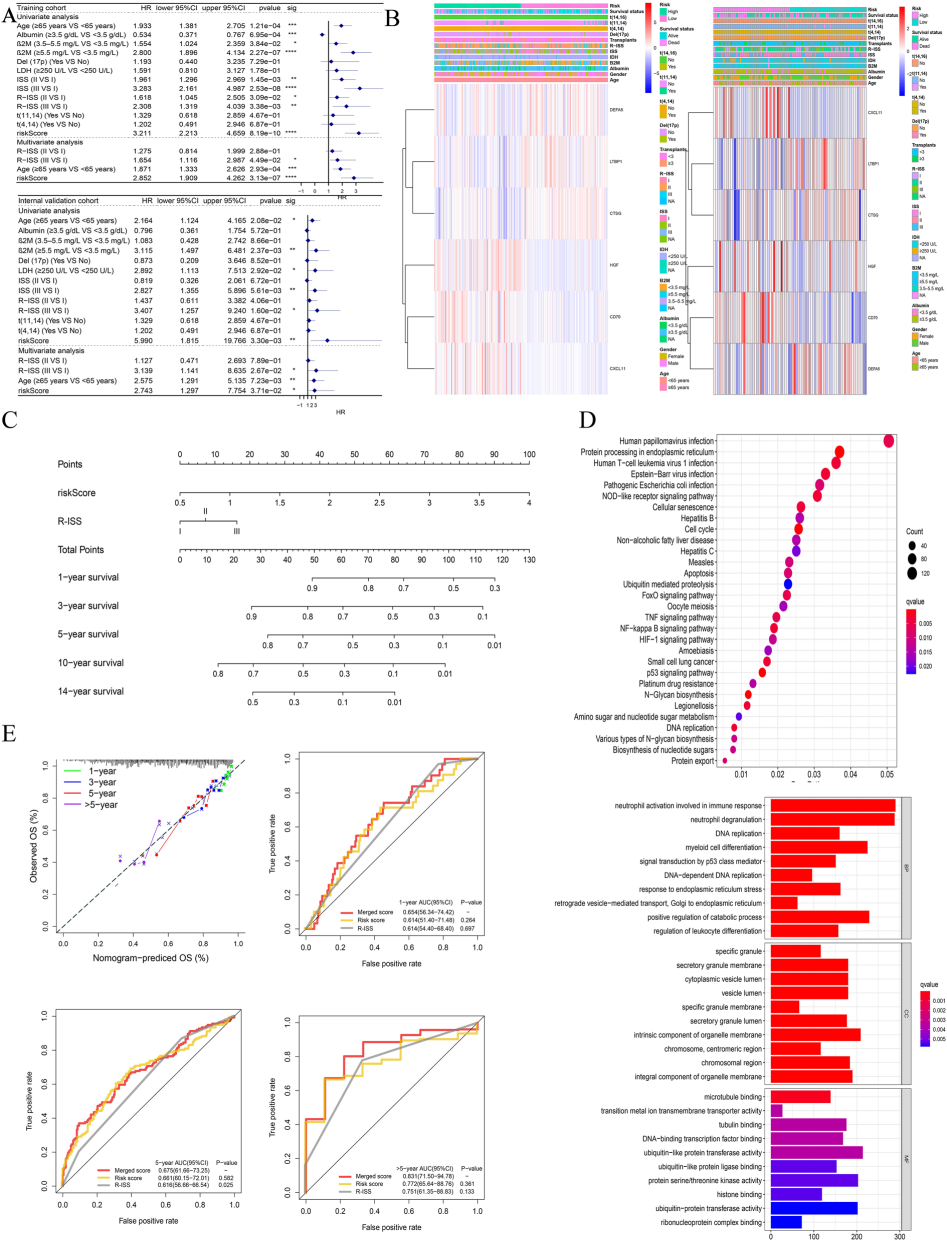
IRGPI is significantly correlated with a variety of clinicopathological factors in MM patients and validates survival prediction. **A** Univariate(top) and Multivariate(bottom) COX analysis in training cohort and internal validation cohort. **B** The heatmap of the IRGPI and clinicopathological characteristics at different risk levels for training cohort and internal validation cohort. Each column showing gene expression or clinicopathological state represents a sample, and each row represents one characteristic or gene in the model. **C** A nomogram was built based on R-ISS and risk score, with calibration plot of the nomogram and time-dependent receiver operating characteristic (ROC) curves of nomograms were compared based on 1-, 5-, and > 5-year OS of the cohort. **D** GO analysis and KEGG pathway analysis shows the top 20 representative pathways in HR in the training cohort (p < 0.05)

To further verify the effect of the model, Kaplan–Meier analyses of survival were performed between groups. Additionally, the sensitivity and specificity of IRGPI were studied via time-dependent receiver operating characteristic (ROC) curve assessment (Fig. 2). Moreover, a nomogram was created to exhibit features for overall survival (OS) integration and visualization, with the consistency index (C-index) and the curve of calibration were employed to assess the nomogram's predictive power (Fig. 3).

Gene ontology (GO) analysis is a common and useful method for annotating genes and products,as well as for identifying characteristic biological attributes of high-throughput genome or transcriptome data [28]. Kyoto Encyclopaedia of Genes and Genomes (KEGG) is a well-known database for systematic analysis of gene functions in biological pathways linking genomic and higher-order functional information [29]. We used the GO and KEGG pathways to reveal potential underlying of the risk score.

The GSEAv4.0.2 software (http://software.broadinstitute.org/gsea/login.jsp) and c2.cp.kegg.v7.0.symbols gene sets were employed to elucidate the physiological pathways associated with the HR and LR patient cohorts. NOM p-value < 0.05 was deemed significant.

### Comprehensive analysis of immune status in HR and LR groups

**Fig. 4:**
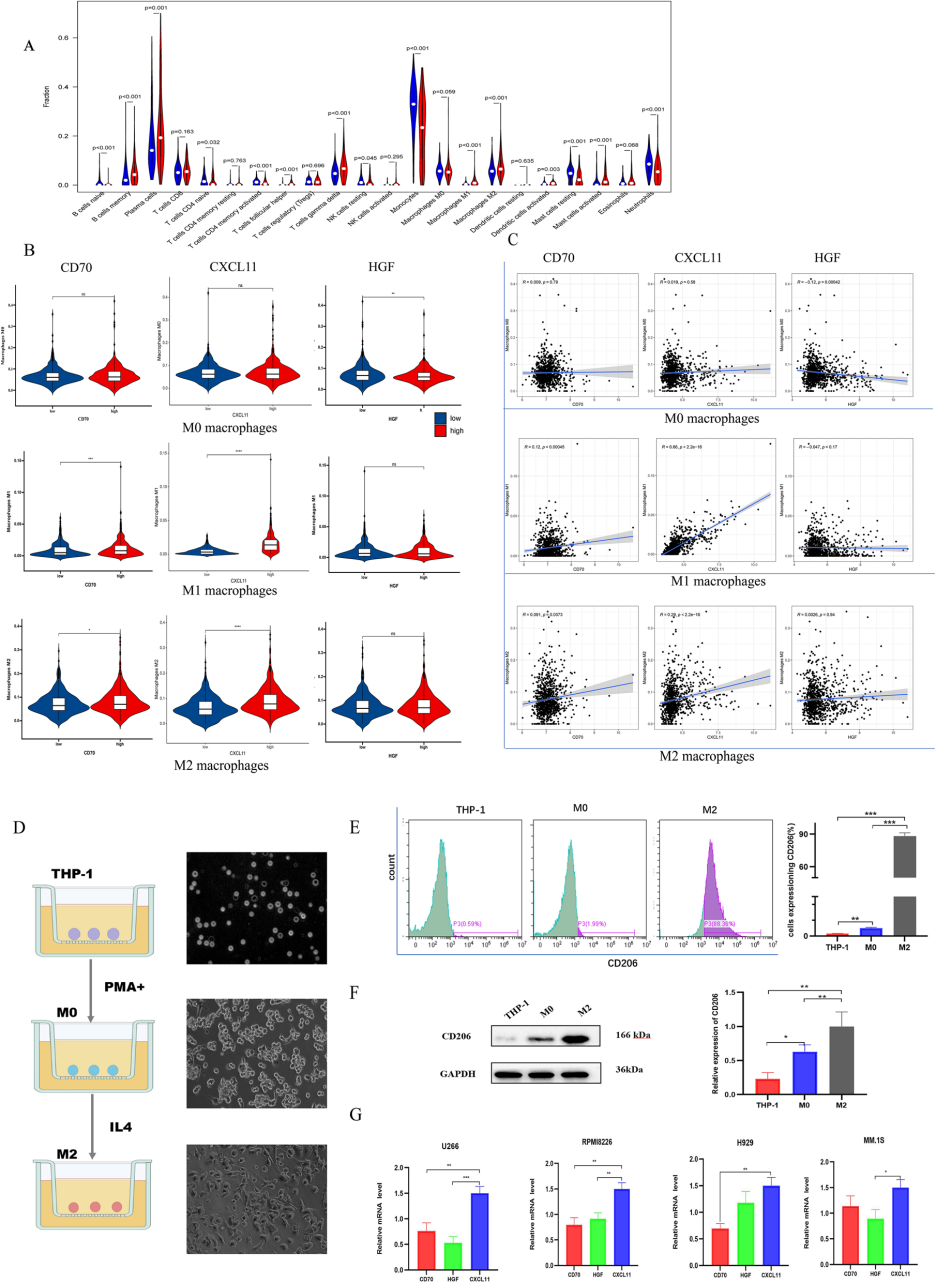
Analyses of immune cell infiltration. **A** Correlations of IRGPI with immune cell infiltration (The blue and red violin represented the IRGPI LR and HR group, respectively. The white points inside the violin implicated median values). **B**, **C** Significant correlations of 3-IRGs (CD70, CXCL11, HGF) with M0 macrophages, M1 macrophages and M2 macrophages in MM. **D** A brief process for M2 macrophage induction. The CD206 expression of M2 macrophages was determined by flow Cytometry (**E**) and Western blot analysis (**F**). **G** qRT-PCR analysis of 3-IRGs after MM cells co-cultured with M2 macrophages. Data were presented as the mean ± SD from three independent experiments. *p < 0.05; **p < 0.01, ***p < 0.001

Based on RNA-sequencing data, immune cells infiltration and the populations of 22 different types of infiltrating immune cells were evaluated using CIBERSORT [26] algorithm (p < 0.05) (Fig. 4).

### Cell culture and co-culture

All cell lines, including THP-1,U266, MM.1S, RPMI8226 and H929 were obtained from the American Type Culture Collection (ATCC, Manassas, VA, USA), and maintained in a 37 °C humid chamber with 5% CO2. RPMI-1640 medium supplemented with 10% fetal bovine serum (FBS) and 1% penicillin/streptomycin was used as a complete cultivation milieu (RPMI 1640, FBS and Pen-Strep were from Gibco). THP-1 cells were activated to form macrophages using 100 nmol/L PMA (Sigma–Aldrich) for 24 h. Then the primary macrophages were stimulated with IL-4 (Sigma-Aldrich) at 20 ng/mL as M2 macrophages. For the coculture assay, MM cells were plated on the upper chamber of 0.4 μm pore transwell inserts (Corning),with PMA-stimulated THP-1 cells plated into the lower chamber.

### Quantitative Real-time PCR

Total RNA was isolated by the TRIzol reagent method (Thermo Fisher Scientific, USA). PrimeScript™ RT Master Mix (Takara Bio, USA) was used for cDNA synthesis, using RNA as a template. RT-qPCR was performed for genes expression analysis using TB Green^®^ Premix Ex Taq (Takara Bio, USA) and The primer sequences were showed in Additional file 6: Table S2. GAPDH was selected as the endogenous control. The comparative Ct method is used to calculate the relative quantification.

### Cell proliferation, apoptosis, transfection and viral infection

Cell Counting Kit-8 (CCK-8) were used to measure cell proliferation. Cells were plated onto a 96-well plate, with 10 µL CCK-8 reagents (Dojindo, Kumamoto, Japan) added to each well. After culture for another 1–2 h, a microplate reader was used tomeasure the absorbance at 450 nm. Cell apoptosis was detected by FITC-Annexin V/PI apoptosis detection kit (KeyGEN, China) in accordance with the manufacturer’s instructions.

For transfection experiments, CXCL11 knockdown (sh-CXCL11) and scrambled control(sh-control) plasmids were constructed by GenePharma Co(Shanghai, China). After transfection of HEK293T cells for 72 h, the viral supernatants were collected and infected MM cells. The viral particles were harvested and concentrated by ultracentrifugation. MM cells were infected with recombinant virus particles in the presence of 6 μg/ml of polybrene (Sigma, USA). Puromycin (2 µg/mL) (Sigma, USA) positively selected infected cells after expansion and maintenance. The transfection efficiency was analyzed by western blotting.

### Immunohistochemical staining (IHC) and flow cytometry

All biopsy specimens were cut into 4 µm sections. For thermally induced epitope repair, a modified citrate buffer was used after dewaxing with xylene. Following blocked with 10% goat serum, the samples were incubated with the specific monoclonal antibody at 4 °C overnight. To determine specific protein expression, modified horseradish peroxidase (HRP) system was used. The specimens were used to perform IHC staining with CD206 antibody (Abcam, ab64693) and CXCL11 antibody (Abcam, ab235977). An electron microscope (Olympus, Tokyo, Japan) was used to acquire high-resolution microphotographs. The corresponding relative integrated optical density (IOD) of protein expression levels in the IHC slices was analyzed using ImageJ software.

Macrophages were co-cultured after 72 h of cultivation and then labeled with FITC-conjugated anti-CD206 antibody (BD, Franklin Lakes, NJ,USA) in accordance with the manufacturer’s instructions prior to detection with flow cytometry.After incubated for 30 min and analyzed with a flow cytometer (Beckman Coulter).

### Western blotting

Cells were lysed with the mixture of RIPA buffer (KeyGEN, China) and protease inhibitor. The BCA method was used to calculate the protein concentration.The extracted proteins were separated on 10% sodium dodecyl sulphate–polyacrylamide gel (SDS-PAGE) and transferred to polyvinylidene fluoride (PVDF) membranes.The primary antibodies included GAPDH (Abcam,ab128915), CD206(Abcam,ab64693), CXCL11(Abcam, ab181035), Bax (CST, 2774), Caspase3 (CST, 9662), followed by goat anti-rabbit IgG H&L (HRP) (Abcam,ab205718). The signal was detected by ECL chemiluminescence detection system (Bio-Rad, USA).

### Xenograft tumor model

BALB/C nude mice (4–5 weeks old, 16–22 g) were used for the experiment in vivo. After randomly assigned into two groups, they were injected with sh-control or sh-CXCL11 U266 cells (1 × 10^7^). The volume of tumors were measured in every 3 days and calculated as follows: volume = (LxW^2^)/2(L and W are the longest and shortest diameters, respectively). The weight of body were also measured.Animal experiment was performed following a protocol approved by the Animal Care and Ethics Committee of Sun Yat-sen University.

## Statistical analysis

Student's t test or a one-way ANOVA was performed for the assessment of continuous variables, and the Fisher exact test or Chisq test was used to assess categorical variables via SPSS computer program version 25 (IBM Corporation, Armonk, NY, USA). R computer program version 3.6.3 (http://www.R-project.org) was employed to conduct statistical assessments. The outcomes were given as mean ± SD of all 3 experiments. All the statistical studies were two-sided, and a p-value cutoff < 0.05 was regarded significant statistically.

## Results

### Patient selection and characteristics

A total of three cohorts, 1207 samples with relevant RNA profile and corresponding clinical information from the GEO database were included in our analysis (Additional file 5: Table S1). The GSE136324 cohort was randomly assigned to two groups: training cohort and internal validation cohort. The GSE57317and GSE4581 were chosen as the external validation cohorts. GSE118985 and the SYSUCC cohorts were used in the validation part. Figure 1 summarizes our research design.

### Identification of a prognostic IRGs signature

First, the survival-associated IRGs were identified by univariate Cox regression in training cohort and validation cohort. Only IRGs with a significant value of p < 0.05 were selected in each cohort. The overlapping showed 11 IRGs (DEFA5, CAMP, CD70, CTSG, CXCL11, HGF, LTBP1, CXCL13, CMTM2, PROK2 and CLEC11A) were identified as survival-associated IRGs. And the interaction of the survival-associated IRGs proteins were summarized alone by STRING (https://string-db.org/) (Additional file 1: Fig. S1A, B).We then explored the mutant variants of the gene panel in CCLE, and the copy number change data were obtained from cBioportal (http://www.cbioportal.org/) [30]. It can be seen that the most common mutation type of these genes is based on gene amplification and deletion. As shown in Additional file 1: Figures S1C.

Finally, the 11 survival-associated IRGs were used to create a prognostic model utilizing the LASSO regression analysis. The IRGPI based on the 6-prognostic gene model was constructed, by the minimum criteria optimal λ value, on the basis of the penalized maximum likelihood estimator of 1000 bootstrap replicates, as shown in Additional file 1: Fig. S1D, E. The following equation was used for this model:

$$\begin{aligned} {\text{Risk score}}\, = & \, - \,0.{\text{14 X DEFA5 level}}\, + \,0.{\text{39 X CD7}}0{\text{ level}}\, - \,0.0{\text{1 XCTSG level}} \\ \, + & \,0.{2}0{\text{ X CXCL11 level}}\, + \,0.{\text{11 XHGF level}}\, - \,0.{2}0{\text{ X LTBP1 level}}{.} \\ \end{aligned}$$

### IRGPI predicts survival of MM patients

According to the formula above, patients were further divided into low-risk (LR) and high-risk (HR) groups employing the median threshold of risk scores. The survival outcome, the risk score, and genes expression profile of patients were studied in Fig. 2. Afterward, the time-dependent ROC curves were used to determine the reliability of IRGPI. In the training cohort, the area under the curve (AUC)for 1-, 3-, 5- and > 5-year survival showed good predictability in Fig. 2B. Furthermore, risk score was evaluated by the same formula mentioned above in the validation cohorts, with the median value of the training cohort's being used as the threshold value. As shown in Fig. 2A, the LR showed a substantially longer OS time and better prognosis than did the HR groups(p < 0.05) in all cohorts.

### Univariate and multivariate Cox analysis

In addition to the metabolic risk score, we comprehensively considered clinical characteristics, such as age, β2-MG, and stage to perform univariate and multivariate Cox regression analyses. This allowed us to determine the significance of the IRGPI on OS. The risk score remained an independent prognostic indicator for OS after adjustment of other clinical confounding variables in multivariate Cox analysis with a hazard ratio of 2.852 [95% CI 1.909–4.262] in the training cohort and 2.743 [95% CI 1.29–7.754] in internal validation cohort (Fig. 3A). Furthermore, the distribution of clinicopathological features and gene expression in different risk groups was shown in Fig. 3B. As expected, patients with higher risk levels were associated with an older age, higher level of β2-MG and higher stage, along with a tendency to poorer survival status. Some of the distribution tendencies are not obvious due to the limited sample size.

### Construction and detection of the predictive nomogram

A nomogram was used for combining the conventional prognostic indicators and the International Staging System (ISS) stage, to provide a more accurate analysis model. To verify the prediction power of the nomogram, the consistency index (C-index) and the calibration curve were employed; the C-index of the nomogram was greater than stage and signature alone,. The AUCs of receiver operating characteristic (ROC) curve created with the combined score, was greater than conventional R-ISS scores and genetic risk category (Fig. 3C). The stability and accuracy of this nomogram can suggest that it might be used in clinical decision-making.

We performed GSEA in training cohort to explore immune-related pathways and other enriched KEGG pathways associated with immune co-variates. Significantly enriched pathways were observed in the HR, most of which were immune-related pathways. Figure 3D showed top 20 representative pathways in HR in training cohort (p < 0.05). In general, immune cascade reaction, immune deficiency, protein processing, T cell receptor signal transduction, virus carcinogenesis and other mechanisms were validated as enriched in HR. Other classical pathways, including cell cycle and cell metabolism, can affect the structural and functional abnormalities of cell components were shown in GO analysis. This provides a theoretical basis for the follow-up mechanism exploration and verification.

### Correlation analysis of immune infiltrating cells in MM microenvironment and identified CXCL11 as a key factor

To further study the possible implications of IRGPI on TME, we focused on the different kinds of invading immune cells in MM. The difference in 22 kinds of tumor-infiltrating immune cells between the LR and HR groups was estimated using CIBERSORT (Fig. 4A). In general, IRGPI is statistically correlated with the extent of immune cells infiltration, which may reflect the status of TME to some level.

In order to identify the key prognostic factor related to prognosis and immune infiltration, we further analyzed the IRGs in the model. The prognostic value of IRGs was further analyzed by Kaplan–Meier after classification as high levels and low levels based on the corresponding optimal cut-off value in the training cohort. As shown in Additional file 2: Fig. S2A–F, the expression of CD70, CXCL11 and HGF was negatively correlated with favorable outcomes, whereas the expression of DEFA5, CTSG and LTBP1 was positively correlated with favorable outcomes. This is consistent with the effect of our model. Among them, the survival differences of CD70, CXCL11 and LTBP1 were statistically significant (*P* < 0.05). Next, we performed ROC analysis for these IRGs as well(Additional file 2: Fig. S2G–L). The AUC values of CD70, CXCL11, HGF, DEFA5, CTSG and LTBP1 was 0.651, 0.684, 0.601, 0.546, 0.400 and 0.337, respectively. Among them, the AUC of HGF, CD70 and CXCL11 are greater than 0.6, which has relatively good predictive significance. This indicated that these three genes have promising diagnostic efficiency for MM.

We then focused on three genes (CD70, CXCL11, and HGF) which negatively correlated with favorable outcomes to further validate the key factor involved in tumor immune infiltration, especially the correlation with macrophages was mainly focused on. The CIBERSORT analysis revealed the umor-associated macrophages (M0, M1, M2) are plotted according to three genes expression level. As shown in Fig. 4B, the level of M2 macrophages was significantly upregulated in the group with high expression of CXCL11 (p < 0.0001) compared to CD70(p < 0.05) and HGF(p < 0.05). Similarly, the CXCL11 expression was strongly correlated with level of M1 macrophages (CXCL11 p < 0.0001, vs CD70 p < 0.05, vs HGF p > 0.05). Figure 4C showed the expression of CXCL11 and its positively correlations with macrophages, particularly M2 (r = 0.29, p < 2.2e−16), while CD70 (r = 0.091, p = 0.0073) and HGF (r = 0.0026, p = 0.94) were not as relevant. Collectively, these data indicated that CXCL11 may affect macrophage polarization and likely enhance immune infiltration cells differentiation.

**Fig. 5:**
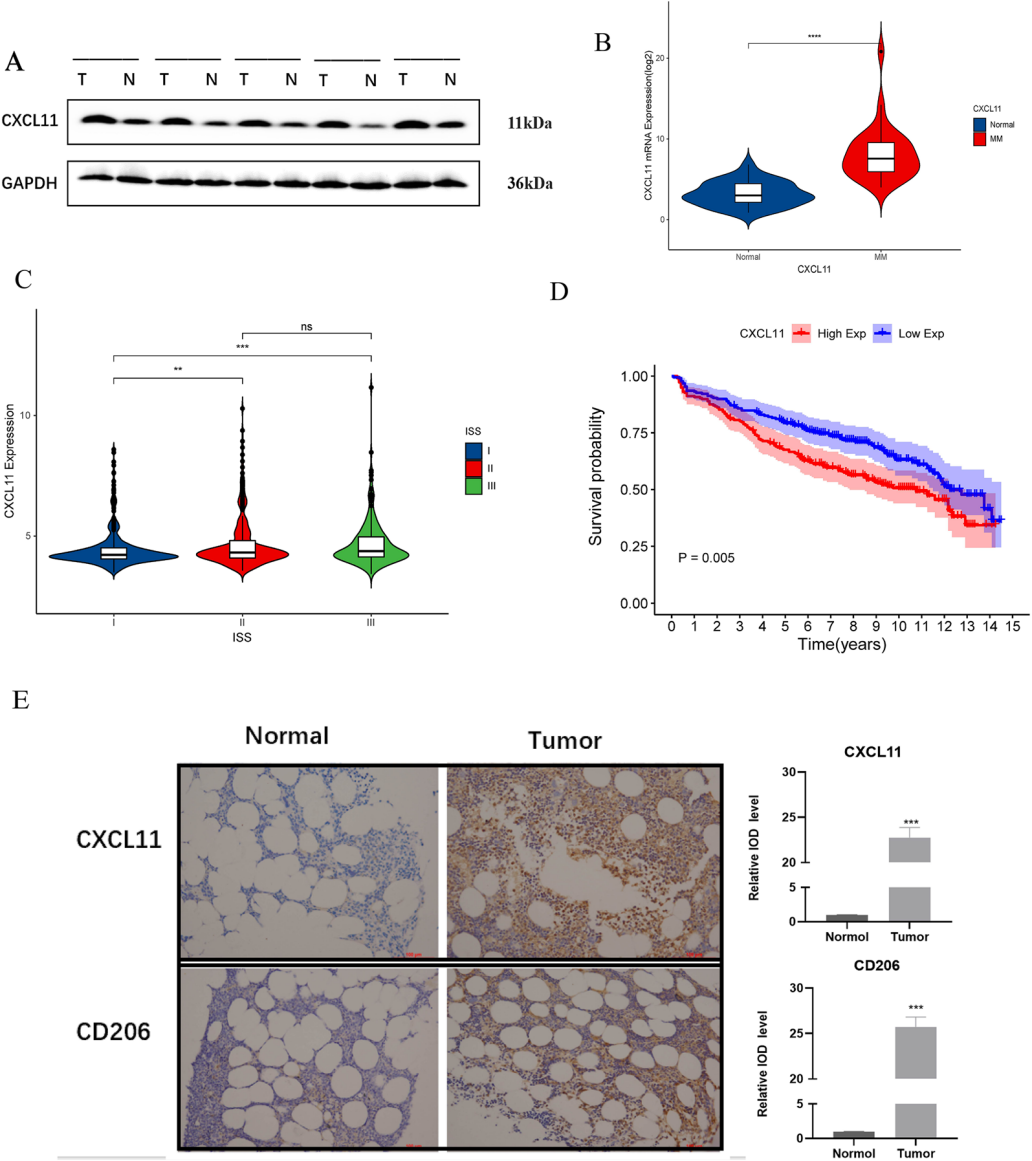
CXCL11 expression is associated with prognosis of MM. **A** Western blot analysis, qRT-PCR analysis (**B**) of CXCL11 expression in MM tissues and normal control. **C** The CXCL11 expression varies with the stage of the patient. **D**The relationship between the expression of overall survival and CXCL11. **E** The images of immunohistochemistry for MM and normal tissues. (The date in **C**, **D** were collected from GSE136324). *p < 0.05; **p < 0.01, ***p < 0.001

Furthermore, we induced M2 macrophages via PMA and IL-4 as the cell model, with the expression of CD206, a marker of M2 polarization for verification (Fig. 4D–F). After co-cultured with M2 macrophages for 48 h, qRT-PCR analysis showed that CXCL11 was the most highly expressed among the 3-IRGs in MM cells (Fig. 4G). This indicated that CXCL11 had significantly correlations with macrophages polarization. Thus, we identified it as a key factor for futher functional tests. We further used the GSE118985 cohort to verify expression levels of the IRGs. The expression of these genes tested in MM tissues was significantly different from that in normal tissues, and CXCL11 was significantly higher (Additional file 3: Fig. S3A). Validation of the expression of 3-IRGs in normal tissues and tumor tissues in our cohort are shown in Additional file 3: Fig. S3B and Fig. 5B.

### CXCL11 expression is increased in MM and associated with poor prognosis in MM patients

To evaluate the expression of CXCL11, we first used qRT-PCR and western blot analysis. The results suggested that the expression of CXCL11 in MM tissues increased compared with non-tumor tissues (Fig. 5A, B). The correlation between CXCL11 overexpression and poor OS was analyzed by Kaplan–Meier based on GSE136324 (Fig. 5D). Figure 5C showed that elevated CXCL11 levels with ISS stage. Immunohistochemistry analysis showed CXCL11 and CD206 was overexpressed in MM tissues as compared with normal tissues (Fig. 5E). These findings verified that CXCL11 might be an oncogenic role and a key factor in macrophages polarization.

### CXCL11 regulates cell proliferation and apoptosis in vitro, associated with macrophage M2-like polarization

**Fig. 6:**
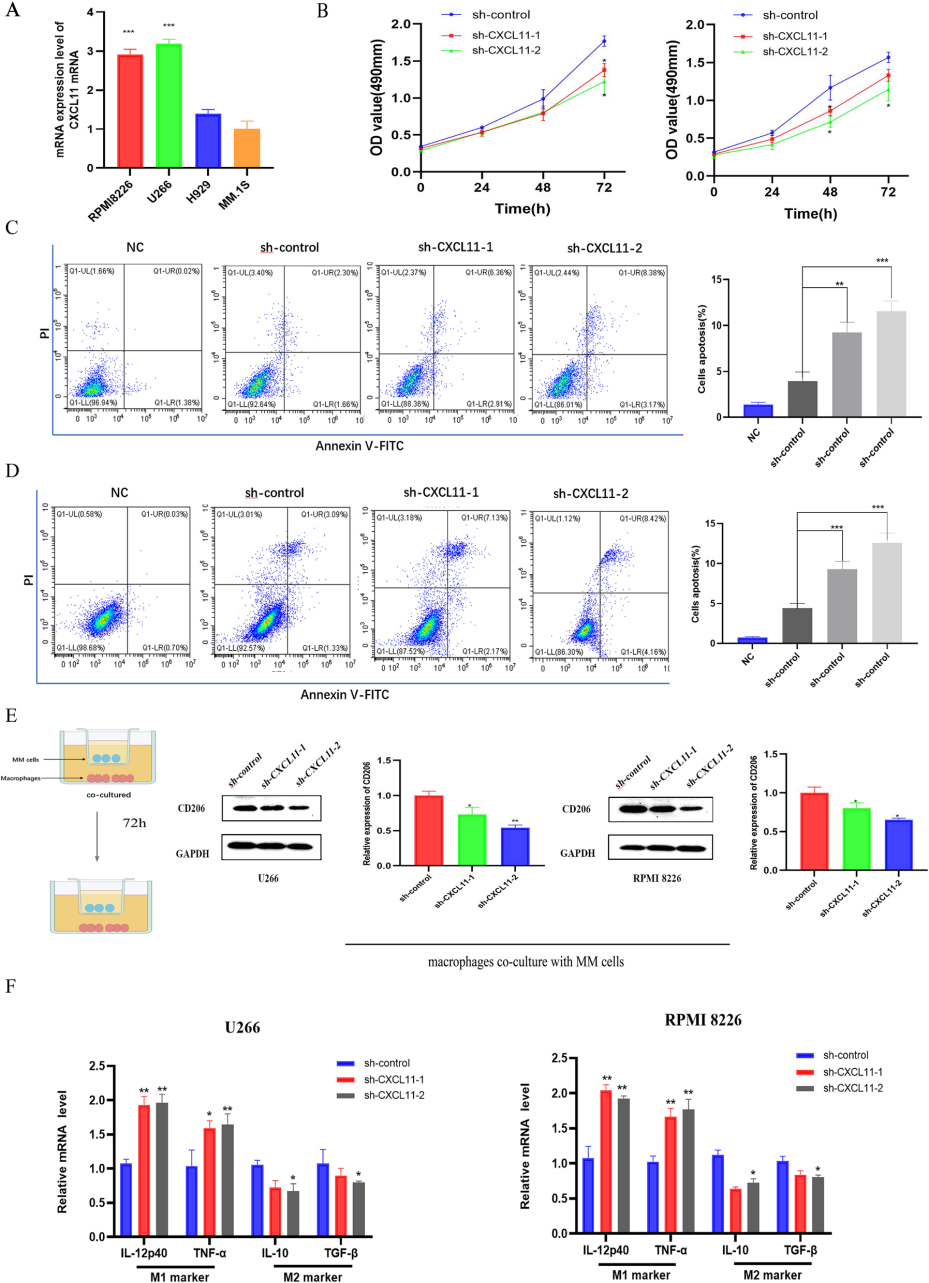
Knock-down of CXCL11 affects MM cells proliferation, apoptosis and macrophages M2-like polarization in vitro. **A** The mRNA expression of CXCL11 in MM cell lines. **B** CCK-8 assays revealed that CXCL11 downregulation decreased MM cell proliferation. **C**, **D**. Apoptosis analysis by flow cytometry in U266 and RPMI 8226 cell lines (C.U266; D.RPMI 8226). **E** CD206 protein expression in macrophages was analyzed by western blot assay at 72 h following co-culture. **F** The expression of M1 and M2 polarization-related markers in macrophages co-cultured with U266 and RPMI 8226 cells transfected with sh-CXCL11 or control was detected by qRT-PCR. Data were presented as the mean ± SD from three independent experiments. *p < 0.05; **p < 0.01, ***p < 0.001

The expression of CXCL11 in myeloma cells was detected by qRT-PCR (Fig. 6A), U266 and RPMI 8226 cells were chosen for the subsequent experiment.We then stably suppressed CXCL11 in the two cell lines by lentiviral transfection. Figure 6B showed the decrease of cell growth rate in sh-CXCL11 group than sh-control group in U266 and RPMI 8226 cells by CCK-8 assays. Flow cytometry analysis revealed a higher percentage of apoptosis in sh-CXCL11 groups than sh-control groups (Fig. 6C, D). Also apoptosis marker detection using western blotting to support the results of flow cytometry to some extent (Additional file 3: Fig. S3C). These data suggested the potential critical roles of CXCL11 on proliferation and apoptosis of MM cells Additional file 4.

We then explored the potential influences of CXCL11 on macrophages using in vitro co-culture system. The expression of CD206, a marker of M2 polarization, detected by western blot analysis after co-cultured 72 h, decreased in sh-CXCL11 groups than those in sh-control groups (Fig. 4E). In addition, qRT-PCR analysis was performed to quantify the expression of M1(IL-12p40, TNF-α) and M2(IL-10, TGF-β) markers. Figure 6F showed that expression of M1 markers and reduced expression of M2 markers in sh-CXCL11 group increased after co-cultured compared with sh-control groups. These findings proposed that CXCL11 may have a potential regulatory role in the M2-like polarization of macrophages.

### CXCL11 promotes macrophage polarization and xenograft growth in vivo

**Fig. 7:**
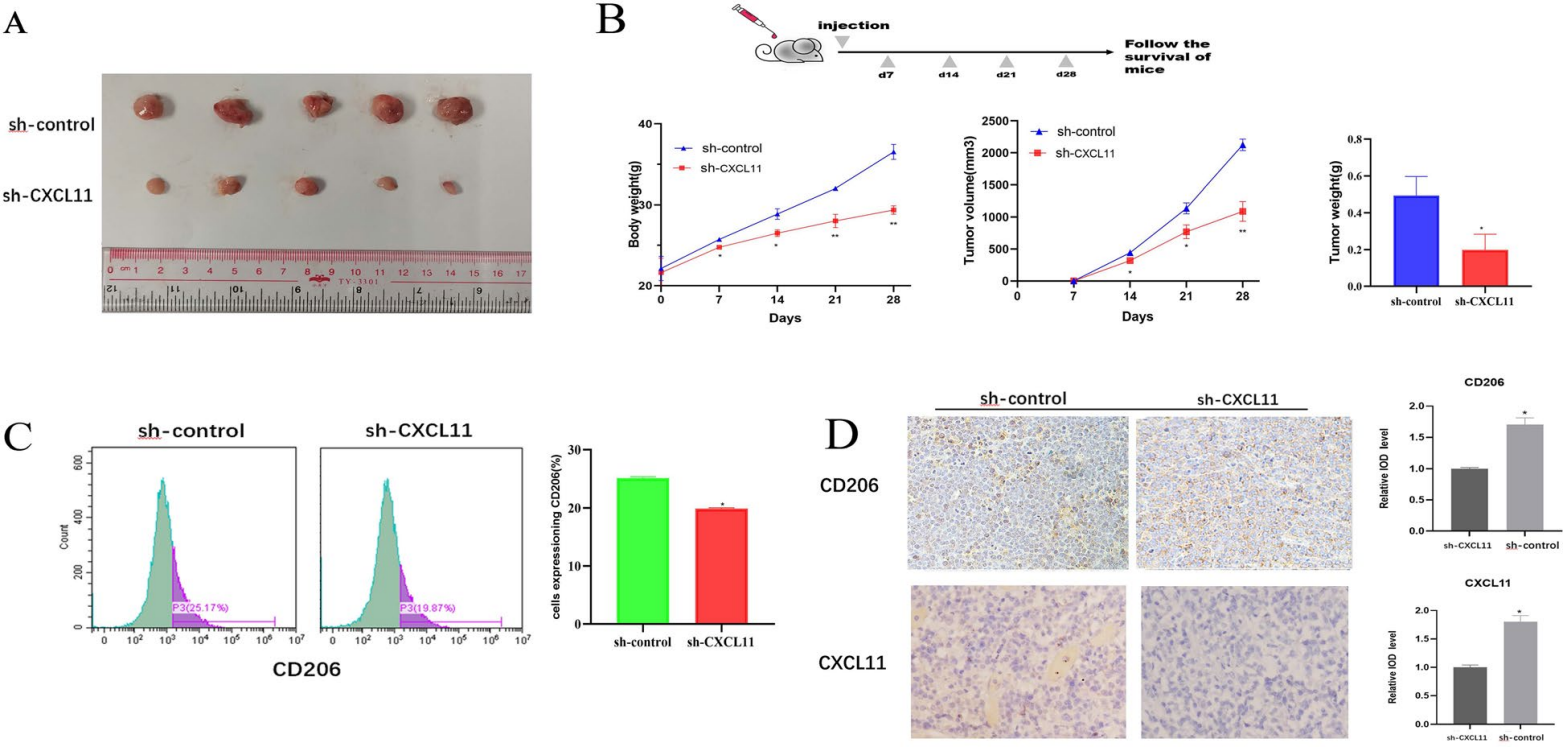
Downregulation of CXCL11 suppressed tumorigenesis in vivo. **A** Representative images of tumors removed from the mice. **B** Body weight and tumor volumes in two groups were observed on the indicated days and tumor weight were shown after removed. **C** Flow cytometry to detect the expression of CD206 in macrophages in primary tumors in the two groups. **D** IHC analysis of CXCL11 and CD206 in primary tumors of two groups. Data were presented as the mean ± SD from three independent experiments. *p < 0.05; **p < 0.01

To study CXCL11 tumor-immune infiltration in the living system, an in vivo model was developed. The CXCL11 stable knockdown or sh-control cells were injected subcutaneously into two groups of nude mice. We assessed the growth of tumor activity and found that the size of tumors in the sh-CXCL11 group were substantially smaller than tumors in the sh-control group (Fig. 7A, B). Moreover, the flow cytometry demonstrated a reduction in the population of infiltrated M2 macrophages in the sh-CXCL11 group (Fig. 7C). Immunostaining revealed that the expression of CXCL11 and CD206 of sh-CXCL11 group was lower than that in control groups (Fig. 7D). These findings supported that knock-down of CXCL11 suppressed tumorigenicity and its involvement in regulating the infiltration of M2 macrophages.

## Discussion

After years of continuous evaluation and progress on various prognostic markers of MM, there is still no effective biomarker to estimate the therapeutic response to immunotherapy and the response to the bone marrow microenvironment [31]. There is evidence that the connection between MM tumors and their microenvironment has a significant role in the progression of MM and the possibility of therapeutic response to immunotherapy [32]. This emphasizes the clinical value of exploring prognostic biomarkers for MM immunotherapy. According to the characteristics of MM and predictable treatment results, exploring more personalized treatments and will help clinical decision-making. Therefore, exploring the IRGPI closely related to the TME of MM can provide potential targets for immunotherapy. It may also increase the accuracy of the prognostic score, detect high-risk patients in a more personalized and specific way, and establish individual treatment strategies for MM patients.

In the present study, we revealed CXCL11 as a regulating factor by using GEO database for analysis and experimental for validation. First, the IRGPI was established based on 6 genes (DEFA5, CD70, CTSG, CXCL11, HGF, LTBP1). which were independent prognostic factors for OS. The model proved to be a valid prognostic immune-related biomarker for MM patients, BDNF is considered to be an effective protective factor to participate in the pathogenesis of glioblastoma [33]. CD70 had been proven to play a role in TME through its effect on human T cells and has become an emerging target for cancer immunotherapy [34, 35]. Studies have shown that CTSG down-regulation was observed in AML patients, and its targeting and inhibition may offer a way for leukemia cells to elude cellular surveillance systems by inhibiting the breakdown of foreign proteins [36], and consistent with our research findings. HGF is believed to promote the various types of tumor formation and progression and may contribute to the either primary or acquired mechanism of resistance to cancer immunotherapy [37, 38]. LTBP1 can be inhibited by other enzymes, leading to the maintenance of tumor cell growth under hypoxic conditions [39]. Our model demonstrates enhanced OS in LR versus HR patients in both training and validation cohorts. A nomogram was also created to estimate survival and validated the model using a time-dependent ROC curve. As compared to alternative staging systems, our findings showed that the model had a higher prognostic value.

Considering the non-negligible role of immune cells infiltration in the myeloma microenvironment, we then used the CIBERSORT method to evaluate the potential of IRGPI to reflect immune cells infiltration. In general, a large number of activated plasma cells are contributed to immune response and related to poor prognosis [40], which is also applied to memory B cells [41]. Among the three genes with promising diagnostic efficiency, CXCL11 is most closely related to the M2 macrophages we focused on. As shown in Fig. 4, the proportion of M2 macrophages in the CXCL11 high expression group was remarkably increased compared with that in the low expression one.Moreover, the level of CXCL11 was most strongly correlated with M2 macrophages. It can be speculated that the high expression of CXCL11 promotes the differentiation of macrophages into M2 macrophages, thereby accelerating the progression of MM. In verification of the gene expression, CXCL11 expressed the highest amount in the simulated co-culture system of MM cells and M2 macrophages. Therefore, we chose it for further functional testing.

CXCL11, a member of the CXC chemokine family, can predict therapeutic sensitivity as potential biomarkers in TME. It performs a task in the regulation of immune cell migration, activation, and differentiation, becoming a new target for immunotherapy [42] and an essential role in a variety of tumors [43–46]. The microenvironment and cells of the bone marrow induce the paracrine or autocrine production of cytokines, promoting the progression of the tumors [42, 43]. Cytokines, in turn, stimulate cancer cell proliferation, differentiation, and apoptosis, establishing a complex dynamic network [44–46]. The microenvironment and chemokines also play a significant interaction in the incidence and progression of MM [47, 48]. Both RNA-sequencing data of GEO database and qRT-PCR detection revealed that high expression of CXCL11 is correlated with poor OS. Functions experiments showed that CXCL11 has oncogenic roles for it drives MM progression in vitro and in vivo. CXCL11 knock-down affected MM cellular proliferation and apoptosis. To futher explore the correlation between CXCL11 and the infiltration of the macrophages, co-cultured model was established, revealing it promote macrophage M2-like polarization. Moreover, M2 macrophage infiltration was abrogated by knockdown of CXCL11 in xenograft tumor. The results verified a substantial correlation between the CXCL11 expression and the infiltration of the macrophages.

This research had some limitations. Firstly, it was a retrospective study with selection bias. The fact that the lacking of the data from some cohorts in validation may affect the prognosis of patients, and the results obtained from large data sets convincingly support our conclusions. In addition, the mechanism of CXCL11 and immune cell recruitment in the TME deserves further study. Looking forward to performing detailed molecular mechanisms and large-scale prospective studies in the future to fully verify our findings.

## Conclusions

In conclusion, we developed a novel prognostic signature according to IRGs. According to the IRGPI, MM patients were categorized into low- and high-risk groups and explored their association with TME. We further identified CXCL11 not only a prognostic indicator but may also reflect immune status by influencing the recruitment of M2 macrophages, promoting tumor cell proliferation. It might have influence on the tumor microenvironment remodeling, thereby affecting the growth of MM. These findings suggested CXCL11 as a novel biomarker with certain value for the prognosis and treatment of MM patients.

## Supplementary Information

**Additional file 1:** **Figure S1.** Construction and validation of the prognostic model. A. Forest plots showing the results of the univariate Cox analysis between gene expression and OS. B. The interactions of survival-associated IRGs proteins.C. Genetic alterations of the IRGs in CCLE, obtained from the cBioportal for Cancer Genomics. D. 100000 bootstrap replicates by lasso regression analysis for variable selection. E. LASSO coefficients of IRGs. Each curve represents one IRG.

**Additional file 2:** **Figure S2.** Kaplan–Meier curve(A-G) and ROC analysis(G-L) of 6-IRGs in the GSE136324 cohort, respectively.

**Additional file 3:** **Figure S3.** Validation of the expression of genes. A. The expression of IRGs in normal and tumor tissues in GSE118985. B. qRT-PCR analysis of two genes (CD70, HGF)expression in tissues.

**Additional file 4:** **Figure S4.** Full images of Western blots.

**Additional file 5:** **Table S1.** The baseline characteristics of patients with multiple myeloma in GEO datasets.

**Additional file 6:** **Table S2.** Primer of experiments.

## Data Availability

The data used and analyzed during the current study are available from the corresponding author on reasonable request.
