# Supplementary figures and images for: Integrative analysis identifies CXCL11 as an immune-related prognostic biomarker correlated with cell proliferation and immune infiltration in multiple myeloma microenvironment
Source: Cancer Cell Int. 2022 May 14;22:187. doi: 10.1186/s12935-022-02608-9 (PMC9107742; doi:10.1186/s12935-022-02608-9)

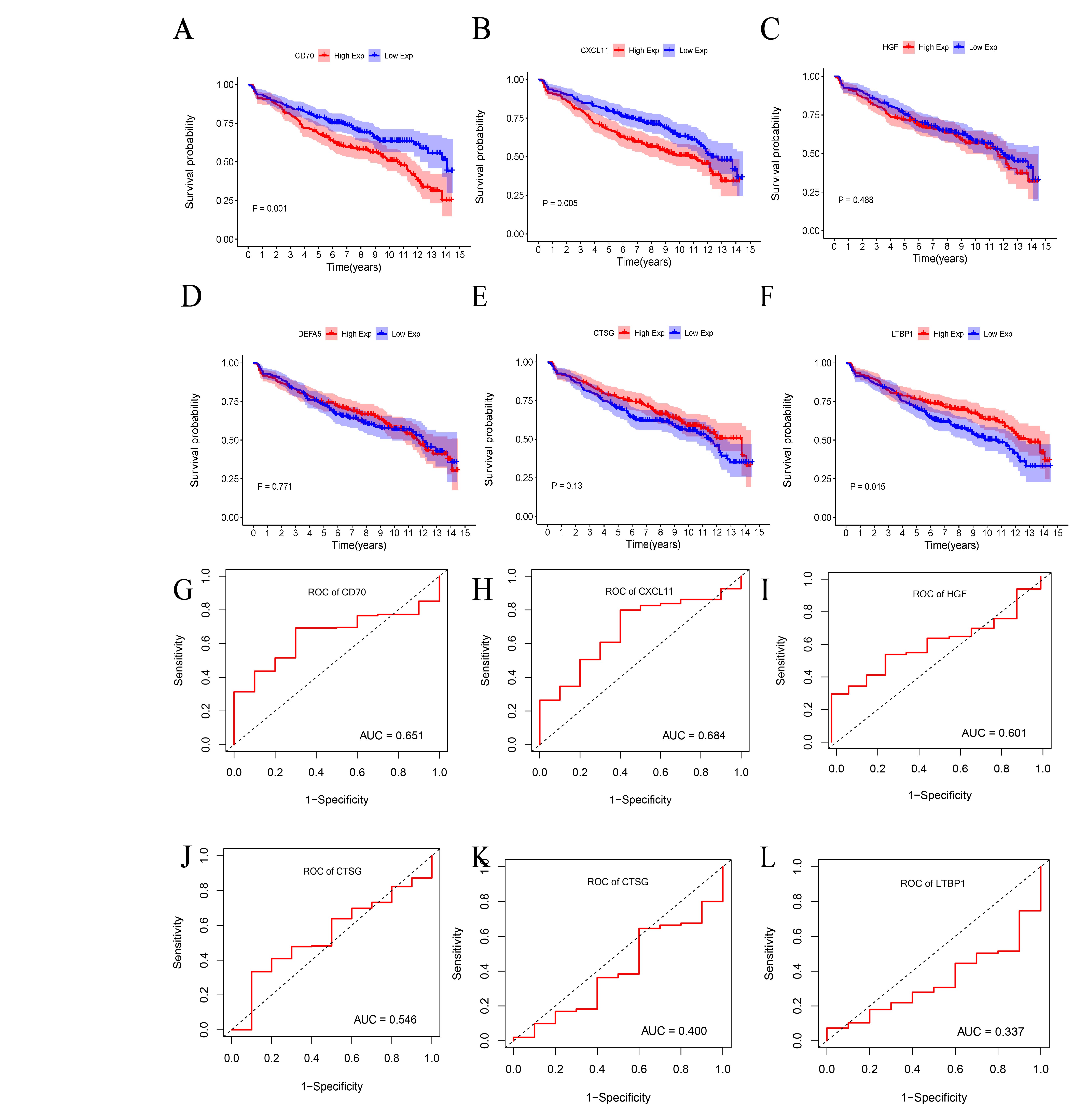

Supplement: Supplementary file 2 — Additional file 2: Figure S2. Kaplan–Meier curve(A-G) and ROC analysis(G-L) of 6-IRGs in the GSE136324 cohort, respectively. [file 12935_2022_2608_MOESM2_ESM.jpg]

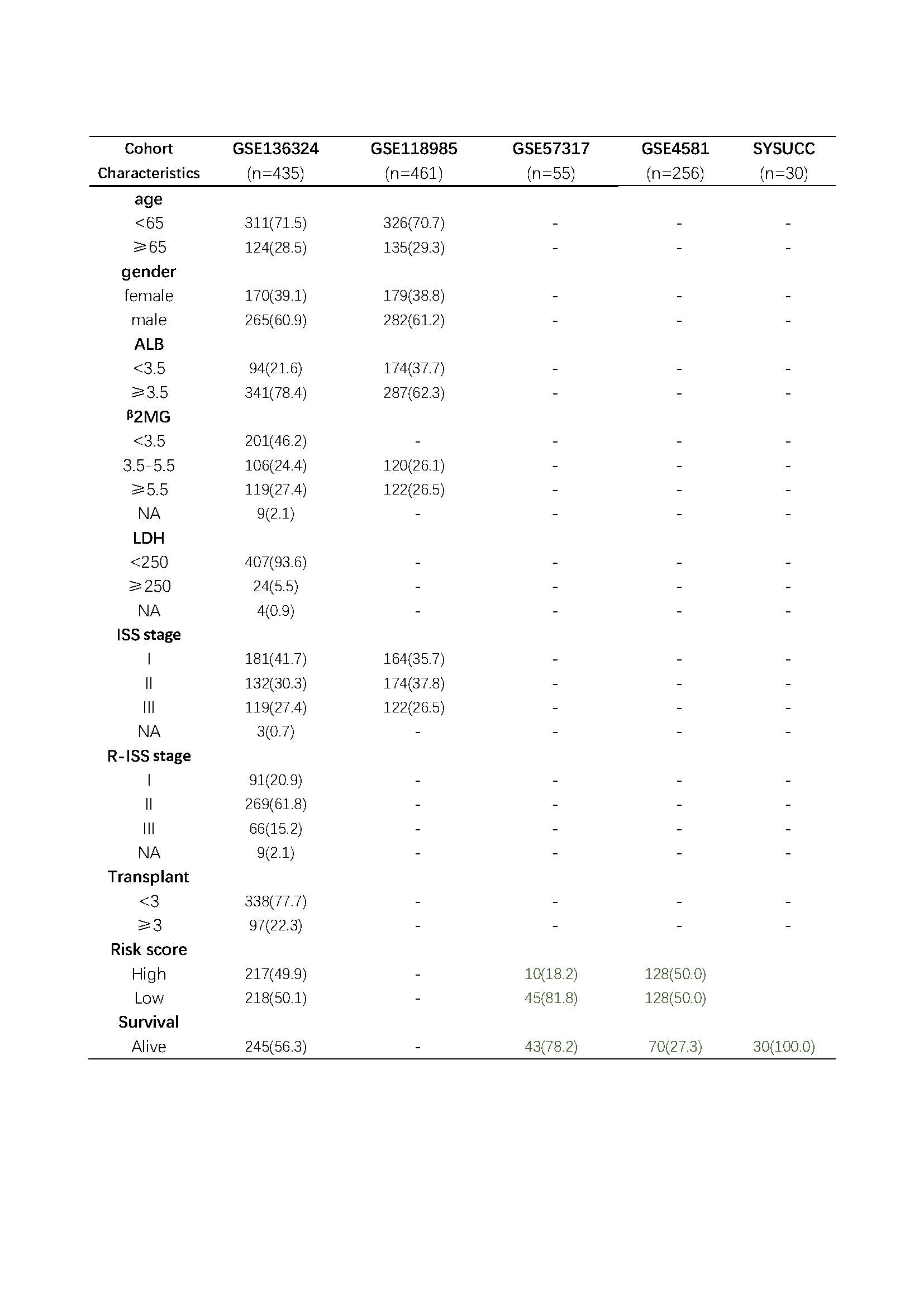

Supplement: Supplementary file 5 — Additional file 5: Table S1. The baseline characteristics of patients with multiple myeloma in GEO datasets. [file 12935_2022_2608_MOESM5_ESM.jpg]

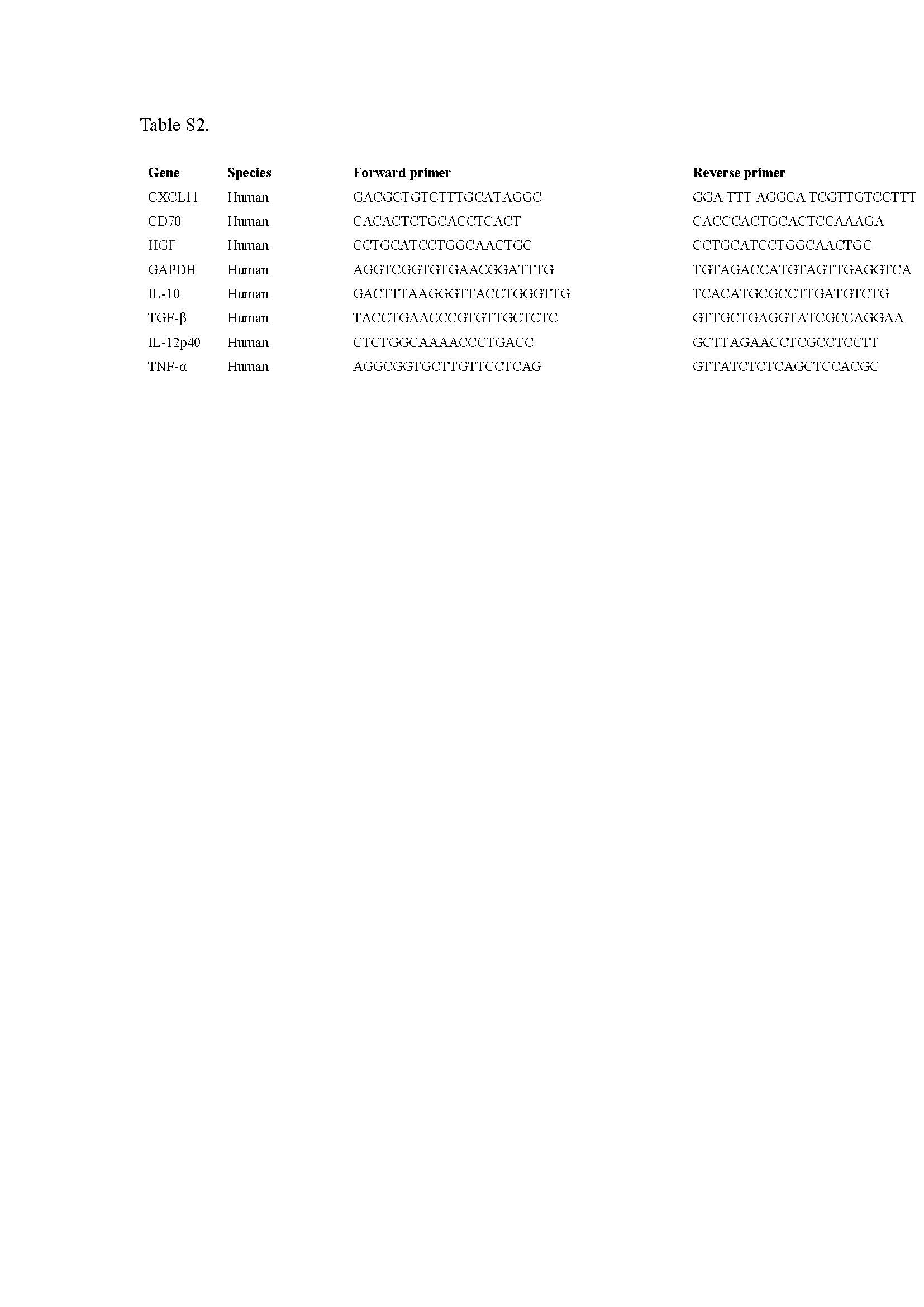

Supplement: Supplementary file 6 — Additional file 6: Table S2. Primer of experiments. [file 12935_2022_2608_MOESM6_ESM.jpg]
